# Methyl carnosate, a carnosic acid derivative, attenuates osteoclastogenesis via modulation of RANKL-induced NF-κB activity

**DOI:** 10.3389/fphar.2026.1825049

**Published:** 2026-06-03

**Authors:** Lifang Zhang, Chengxu Xie, Xinyi Bao, Mojtaba Tabandeh, Vishwa Deepak

**Affiliations:** 1 Osteoimmunology and Drug Discovery Research Group, Department of Biology, College of Science, Mathematics and Technology, Wenzhou-Kean University, Wenzhou, Zhejiang, China; 2 Dorothy and George Hennings College of Science, Mathematics and Technology, Kean University, Union, NJ, United States; 3 Department of Chemistry, College of Science, Mathematics and Technology, Wenzhou-Kean University, Wenzhou, Zhejiang, China; 4 International Frontier Interdisciplinary Research Institute (IFIRI) of Wenzhou-Kean University, Wenzhou, Zhejiang, China; 5 Wenzhou Municipal Key Laboratory for Applied Biomedical and Biopharmaceutical Informatics, Wenzhou-Kean University, Wenzhou, Zhejiang, China; 6 Zhejiang Bioinformatics International Science and Technology Cooperation Center, Wenzhou-Kean University, Wenzhou, Zhejiang, China; 7 Zhejiang-Malaysia Joint Laboratory For Rare Medicinal Resources, Wenzhou-Kean University, Wenzhou, Zhejiang, China

**Keywords:** bone resorption, methyl carnosate, NF-κB, osteoclastogenesis, RANKL

## Abstract

Excessive osteoclast activity underlies bone-destructive diseases including osteoporosis and rheumatoid arthritis. RANKL-induced NF-κB signaling represents a critical pathway driving osteoclastogenesis. Here, we report that methyl carnosate, a naturally occurring diterpene from rosemary, inhibits RANKL-induced osteoclastogenesis with an IC_50_ of 1.2 μM (95% CI: 0.9521–1.372 μM) and displays greater potency than its parent compound, carnosic acid. Promoter–reporter analysis further indicates attenuation of RANKL-induced NF-κB activity, consistent with downstream suppression of NFATc1 mRNA expression. These findings identify methyl carnosate as a promising pharmacological candidate for the development of bone-protective therapeutics.

## 1 Introduction

Dysregulated osteoclast activity drives bone loss in osteoporosis, inflammatory arthritis, and bone metastases (Takegahara et al., 2024). RANKL-induced NF-κB signaling is essential for osteoclast differentiation, making it an attractive therapeutic target (Zhou et al., 2022). Current anti-resorptive agents have limitations including adverse effects, highlighting the need for novel therapeutics (Naciu et al., 2025).

Carnosic acid, a phenolic diterpene from rosemary, inhibits RANKL-induced osteoclastogenesis at concentrations of 5–15 μM. However, the relatively high effective doses and limited stability may constrain its therapeutic potential (Thummuri et al., 2017; Zheng et al., 2020). Methyl carnosate, a naturally occurring methyl ester derivative of carnosic acid, exhibits enhanced antioxidant activity and stability in lipid systems (Climati et al., 2013; Huang et al., 1996). However, its effects on osteoclastogenesis remain unexplored. Given that esterification typically enhances cellular permeability, we hypothesized that methyl carnosate might exhibit improved anti-osteoclast potency. Here we investigated whether methyl carnosate inhibits RANKL-induced osteoclast differentiation and assessed its mechanism of action.

## 2 Materials and methods

### 2.1 Reagents and cell culture

Methyl carnosate (CAS 82684-06-8, purity ≥98%, MedChemExpress, NJ, USA) was dissolved in DMSO (10 mM stock, final concentration ≤0.1%). RAW 264.7 murine macrophages were cultured in DMEM with 10% FBS at 37 °C, 5% CO_2_. For Osteoclast differentiation was performed as previously described (Deepak et al., 2022). Briefly cells were treated with 50 ng/mL recombinant murine RANKL (R&D Systems, MN, USA) ± methyl carnosate for 5 days.

### 2.2 Cell viability and TRAP activity assays

Cell viability was assessed using CCK-8 assay (0.1–10 μM methyl carnosate, 48–72 h). TRAP activity was measured using a colorimetric kit (#EEA055, ThermoFisher Scientific, MA, USA) following osteoclast differentiation. IC_50_ values were calculated using GraphPad Prism 9.

### 2.3 NF-κB reporter assay

RAW 264.7 cells stably expressing NF-κB-luciferase reporter (#D2206, Beyotime, China) were pre-treated with vehicle or methyl carnosate (1 h), then stimulated with RANKL (6 h). Luciferase activity was measured using Steady-Glo® system (Promega, WI, USA).

### 2.4 RT-qPCR analysis

Total RNA was extracted from RANKL-stimulated RAW 264.7 cells in the presence or absence of methyl carnosate (1.2 μM) for 3-days. cDNA was synthesized from 1 μg RNA, and quantitative real-time reverse transcription polymerase chain reaction (qRT-PCR) was performed on a LightCycler® 96 system (Roche, Basel, Switzerland). Relative gene expression was calculated using the 2^−ΔΔCT^ method with *GAPDH* as the internal control. Primer sequences: *NFATc1* (NCBI Gene ID 18018; F: 5′-GAC​CCG​GAG​TTC​GAC​TTC​G-3′, R: 5′-TGA​CAC​TAG​GGG​ACA​CAT​AAC​TG-3′; amplicon 97 bp); *GAPDH* (NCBI Gene ID: 14433; F: 5′-AGG​TCG​GTG​TGA​ACG​GAT​TTG-3′, R: 5′-TGT​AGA​CCA​TGT​AGT​TGA​GGT​CA-3′; amplicon 123 bp).

### 2.5 Statistical analysis

Data represent mean ± SD from N = 3 independent experiments unless otherwise stated. Concentration–response data were fitted using a four-parameter logistic (4 PL) model; data points represent mean ± 95% CI. Statistical significance was determined by one-way ANOVA with Tukey’s *post hoc* test (*p* < 0.05 considered significant).

## 3 Results

### 3.1 Methyl carnosate is well-tolerated in osteoclast precursor cells

**FIGURE 1:**
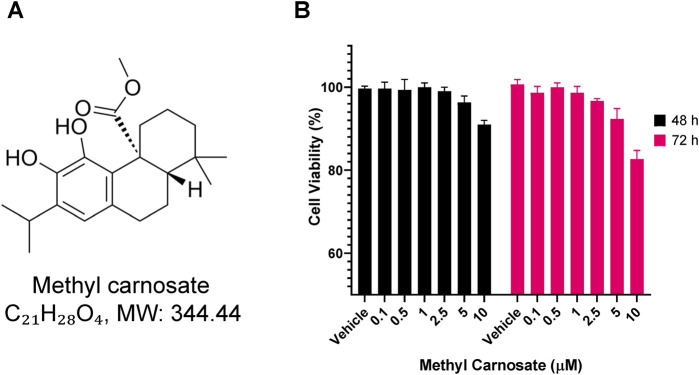
Chemical structure and cytotoxicity profile of methyl carnosate. **(A)** Chemical structure of methyl carnosate (C_21_H_28_O_4_, MW: 344.44). **(B)** Cell viability of RAW 264.7 cells treated with indicated concentrations of methyl carnosate for 48 h (black bars) or 72 h (pink bars), measured by CCK-8 assay. Data represent mean ± SD, N = 3 independent experiments.

The chemical structure of methyl carnosate, a naturally occurring methyl ester of carnosic acid, is shown in Figure 1A. RAW 264.7 macrophages were treated with increasing concentrations of methyl carnosate (0.1–10 μM) for 48 and 72 h. Cell viability remained >95% at concentrations up to 5 μM at both time points, with viability >80% even at 10 μM (Figure 1B). These results established a favorable safety window and confirmed that concentrations up to 5 μM could be used in subsequent osteoclastogenesis assays without confounding cytotoxic effects.

### 3.2 Methyl carnosate potently inhibits RANKL-induced osteoclastogenesis and suppresses NF-κB signaling

**FIGURE 2:**
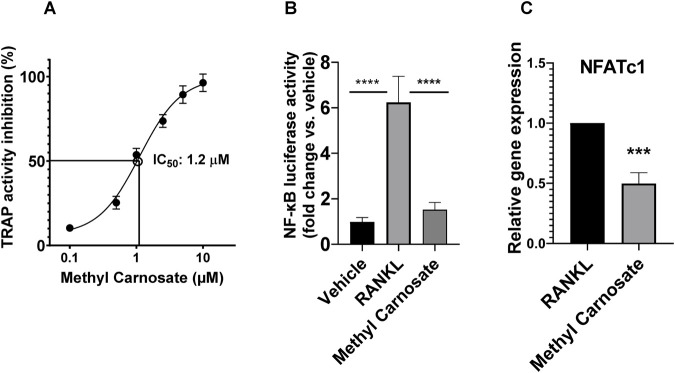
Methyl carnosate inhibits RANKL-induced osteoclastogenesis, suppresses NF-κB activity, and reduces NFATc1 expression. **(A)** Dose-dependent inhibition of TRAP activity in RANKL-stimulated RAW 264.7 cells by methyl carnosate. IC_50_ = 1.2 μM (95% CI: 0.9521–1.372 μM; 4 PL regression). Data points represent mean ± 95% CI, N = 3 independent experiments. **(B)** Effect of methyl carnosate on RANKL-induced NF-κB transcriptional activity measured by luciferase reporter assay. Bars represent mean ± SD, N = 3 independent experiments. ****p < 0.0001 vs. RANKL alone (one-way ANOVA with Tukey’s *post hoc* test). **(C)** NFATc1 relative mRNA expression in RANKL-stimulated RAW 264.7 cells treated with methyl carnosate (1.2 μM) or vehicle control, normalized to GAPDH. Bars represent mean ± SD, N = 3 independent experiments. ***p < 0.001 vs. control (one-way ANOVA with Tukey’s *post hoc* test).

To evaluate anti-osteoclastogenic activity, RAW 264.7 cells were stimulated with RANKL in the presence of varying concentrations of methyl carnosate. The compound dose-dependently inhibited RANKL-induced TRAP activity with an IC_50_ of 1.2 μM (95% CI: 0.9521–1.372 μM; 4 PL regression, N = 3; Figure 2A), demonstrating approximately 4–12-fold greater activity than previously reported potency of its parent compound carnosic acid (IC_50_ 5–15 μM) (Thummuri et al., 2017; Zheng et al., 2020). This substantial improvement in potency likely reflects enhanced cellular permeability conferred by increased lipophilicity of the methyl ester.

To elucidate the mechanism, we examined NF-κB signaling using a luciferase reporter assay. RANKL stimulation induced robust NF-κB activation (∼6-fold over vehicle control), whereas methyl carnosate pre-treatment reduced this activation to near-basal levels (Figure 2B), demonstrating significant interference with the NF-κB signaling cascade essential for initiating the osteoclastogenic transcriptional program (Asagiri and Takayanagi, 2007). Consistent with NF-κB suppression, methyl carnosate significantly reduced NFATc1 mRNA expression compared to RANKL-stimulated controls (Figure 2C, N = 3, ***p < 0.001), providing direct transcriptional evidence of downstream pathway inhibition.

## 4 Discussion

This study identifies methyl carnosate as a potent inhibitor of RANKL-induced osteoclastogenesis, exhibiting an IC_50_ of 1.2 μM, representing approximately 4–12-fold greater potency than its parent compound carnosic acid. The IC_50_ lies well below cytotoxic concentrations, indicating a favorable therapeutic window and specific pathway inhibition rather than general cytotoxicity.

The markedly enhanced potency of methyl carnosate relative to carnosic acid is noteworthy. Esterification may confer increased lipophilicity and enhanced membrane permeability, and potentially improved metabolic stability (Huang et al., 1996); however, formal ester hydrolysis and stability assays are required to confirm the structural basis for the enhanced potency. These findings position methyl carnosate as a promising pharmacological candidate within the carnosic acid chemotype.

Mechanistically, methyl carnosate significantly suppressed RANKL-induced NF-κB transcriptional activity, reducing activation from ∼6-fold to near-basal levels. NF-κB activation is a critical early event in osteoclastogenesis, required for induction of NFATc1, the master transcription factor driving osteoclast differentiation (Asagiri and Takayanagi, 2007). Consistent with these findings, *NFATc1* mRNA was significantly suppressed (∼50%) by methyl carnosate treatment, providing direct transcriptional validation of NF-κB pathway inhibition. Suppression of NF-κB signaling by methyl carnosate thus provides a coherent mechanistic basis for its potent anti-osteoclastogenic activity. Taken together, these findings establish methyl carnosate as a promising NF-κB modulator and a potential pharmacological candidate for developing novel bone-protective therapeutics.

Several limitations of this study should be noted. First, in this study RAW 264.7 murine macrophages, a widely used and validated pharmacological screening model for RANKL-induced osteoclastogenesis were utilized. Further validation in primary bone marrow macrophages and human monocyte-derived osteoclasts will be necessary to establish physiological and translational relevance. Second, the structural basis for the enhanced potency relative to carnosic acid remains to be confirmed by formal ester hydrolysis and metabolic stability studies. Third, PK/PD characterization, including bioavailability and tissue distribution, will be essential before *in vivo* translation.

Future studies should investigate effects on additional RANKL-activated cascades including MAPK signaling and validate downstream transcription factor modulation (c-Fos, NFATc1 protein levels). Demonstration of bone-protective efficacy in standard *in vivo* models, including the ovariectomy-induced osteoporosis and collagen-induced arthritis mouse models, represents the critical next experimental objective for this compound.

## Data Availability

The original contributions presented in the study are included in the article/supplementary material, further inquiries can be directed to the corresponding author.
